# Intestinal disturbances associated with mortality of children with complicated severe malnutrition

**DOI:** 10.1038/s43856-023-00355-0

**Published:** 2023-09-29

**Authors:** Bijun Wen, Amber Farooqui, Celine Bourdon, Nawar Tarafdar, Moses Ngari, Emmanuel Chimwezi, Johnstone Thitiri, Laura Mwalekwa, Judd L. Walson, Wieger Voskuijl, James A. Berkley, Robert H. J. Bandsma

**Affiliations:** 1 Department of Nutritional Sciences, Faculty of Medicine, University of Toronto, Toronto, Canada; 2 Department of Translational medicine, Hospital for Sick Children, Toronto, Canada; 3 The Childhood Acute Illness & Nutrition Network, Nairobi, Kenya; 4 KEMRI/Wellcome Trust Research Programme, Kilifi, Kenya; 5 Department of Paediatrics, Coast General Hospital, Mombasa, Kenya; 6 Departments of Global Health, Medicine, Pediatrics and Epidemiology, University of Washington, Seattle, WA USA; 7 Amsterdam Institute for Global Health and Development, Department of Global Health, Amsterdam University Medical Centres, Amsterdam, The Netherlands; 8 Department of Paediatrics and Child Health, Kamuzu University of Health Sciences (formerly College of Medicine), Blantyre, Malawi; 9 Centre for Tropical Medicine & Global Health, Nuffield Department of Medicine, University of Oxford, Oxford, United Kingdom; 10 Department of Biomedical Sciences, Kamuzu University of Health Sciences (formerly College of Medicine), Blantyre, Malawi

**Keywords:** Paediatric research, Gastrointestinal diseases, Malnutrition

## Abstract

**Background:**

Children admitted to hospital with complicated severe malnutrition (CSM) have high mortality despite compliance with standard WHO management guidelines. Limited data suggests a relationship between intestinal dysfunction and poor prognosis in CSM, but this has not been explicitly studied. This study aimed to evaluate the role of intestinal disturbances in CSM mortality.

**Methods:**

A case-control study nested within a randomized control trial was conducted among children hospitalized with CSM in Kenya and Malawi. Children who died (cases, *n* = 68) were compared with those who were discharged, propensity matched to the cases on age, HIV and nutritional status (controls, *n* = 68) on fecal metabolomics that targeted about 70 commonly measured metabolites, and enteropathy markers: fecal myeloperoxidase (MPO), fecal calprotectin, and circulating intestinal fatty acid binding protein (I-FABP).

**Results:**

The fecal metabolomes of cases show specific reductions in amino acids, monosaccharides, and microbial fermentation products, when compared to controls. SCFA levels did not differ between groups. The overall fecal metabolomics signature moderately differentiates cases from controls (AUC = 0.72). Enteropathy markers do not differ between groups overall, although serum I-FABP is elevated in cases in a sensitivity analysis among non-edematous children. Integrative analysis with systemic data suggests an indirect role of intestinal inflammation in the causal path of mortality.

**Conclusions:**

Intestinal disturbances appear to have an indirect association with acute mortality. Findings of the study improve our understanding of pathophysiological pathways underlying mortality of children with CSM.

## Plain Language Summary

Malnourished children are at a high risk of dying when exposed to an acute illness. They often have symptoms like diarrhea that indicate their gut is not working properly. It is unclear whether these gut problems contribute to their deaths. Feces contain numerous small molecules processed by the gut that reflect gut health. We compare these fecal molecules between malnourished children who died during hospitalization to those who survived, and relate them to signs of inflammation in the body. We show that the fecal molecules are different between children who died and those who survived. These differences reveal that poor gut health could increase risk of death, potentially by perturbing the body’s defensive response to an acute illness. These findings underscore that treatment for ill severely malnourished children should focus on improving gut health.

## Abstract (web summary)

Wen et al. investigate associations between intestinal disturbances and mortality in children hospitalized with complicated severe malnutrition. Differences are seen in the fecal metabolome of children who die compared with those who are discharged, with integrative analyses suggesting an indirect role for intestinal inflammation in mortality.

## Introduction

In-hospital mortality among children with severe malnutrition remains high despite strict adherence to therapeutic and refeeding protocols recommended by the World Health Organization (WHO). This population is commonly referred to as severe malnutrition (SAM), and more recently as severe malnutrition (SM) to encompass both the chronic and acute aspects of the condition^1,2^. When severely malnourished children have concurrent serious illnesses (e.g., acute infections or other complications), they are at higher risks of dying and require hospital-based management compared to those without serious illnesses. Hence, children hospitalized with severe malnutrition are ‘complicated’ cases, and are referred to as complicated severe malnutrition (CSM)^3^. Some evidence suggests that diarrhea, reflecting intestinal dysfunction, is associated with an increased risk of death among these children^4–6^, suggesting a link between intestinal microenvironment disturbances and mortality.

Pathophysiological changes in the intestine, characterized by mucosal atrophy, barrier dysfunction, intestinal inflammation, and nutrition malabsorption, are commonly seen in malnourished children living in low- and middle-income countries^7,8^. These subclinical intestinal changes have been linked to increased bacterial translocation across the intestinal lumen leading to systemic inflammation^9^. The intestinal microbiota plays a pivotal role in modulating intestinal homeostasis and host metabolism^10^. This is mediated by an active microbial metabolic repertoire that produces numerous biologically active substances, such as short-chain fatty acids (SCFAs), vitamins, secondary bile acids, phenolics, aromatic acids, lipids, and neurotransmitters^11^. Indeed, the fecal metabolome has been considered as a functional readout of microbial activity of the intestine. The microbiota of severely malnourished children has been reported to have reduced diversity and persistent immaturity compared to well-nourished children^12–14^. In mouse models of malnutrition, compositional changes of the microbiota were accompanied by shifts in the profile of fecal metabolites, including those with potentially important health implications such as SCFAs and amino acids^15,16^. A partial causal relationship between gut microbial disturbances and the development of enteropathy and malnutrition phenotypes was demonstrated in these preclinical studies^15–17^.

The enteropathy observed in children with malnutrition conceivably puts them at a greater risk of life-threatening infections and complications. In critically ill well-nourished children, an association between intestinal disturbances and mortality has been shown^18–21^. Severely malnourished children are vulnerable to diarrhea-related dehydration, electrolyte imbalances, and gram-negative bacteremia^22–24^. These common complications point towards an intestinal origin, suggesting that intestinal dysfunction may cause mortality through infectious diarrhea or enteric pathogen-induced bacteremia. Yet, scarce data are available on the role of intestinal disturbances in the mortality of CSM. A previous case-control study from our group showed that although diarrhea, increased intestinal inflammation, and reduced fecal SCFAs did not have a direct effect on mortality, they were associated with increased systemic inflammation, which was directly associated with mortality^25^. However, this was a relatively small study that examined a few fecal analytes, which may not provide a comprehensive representation of the intestinal microenvironment, nor the complexity of enteropathy seen in these children. It remains unclear whether and how intestinal disturbances may contribute to inpatient mortality of CSM. The interplay between intestinal enteropathy, microbiota activity, systemic factors and mortality needs to be investigated to a greater depth. The current study examined the association between intestinal disturbances and mortality in CSM by comparing the fecal metabolome and a set of enteropathy markers at hospital admission of children who subsequently died to those survived. Differences are seen in the fecal metabolome of children who die compared with those who are discharged, and intestinal inflammation appears to have an indirect association with mortality.

## Methods

### Study design and patients

This was a nested case-control study among children with CSM enrolled to a multicenter randomized controlled trial (NCT02246296) as described in detail by Bandsma et al.^26^. The current study addresses some of the pre-specified secondary outcomes of the parent trial. Children with CSM were defined as those with MUAC < 11.5 cm, or WHZ < −3 (age 6 to 59 months, BMI-for-age Z-score < −3 (age ≥ 60 months), or oedematous malnutrition (at any age), and having medical complications or failed an appetite test according to the WHO guidelines^3^. This case-control study used fecal samples collected from participants on admission (before randomization and treatment initiation). Cases (nonsurvivors: NS) were children who died during hospitalization, while controls (survivors: S) were children discharged alive within 14 days of hospitalization and matched to the cases by propensity score for mortality based on age, MUAC, and HIV status^27^. The sample size was limited by sample availability. All available samples collected from NS (*n* = 68) were included, which represents 54% of all death cases in the parent trial. S were matched with NS at a 1:1 ratio. Sampling procedures are shown in Supplementary Fig. 1. Baseline patient characteristics were summarized as medians with interquartile ranges (IQRs), mean ± the standard deviations for continuous variables, or percentages for categorical variables. Patient and public involvement was not part of this study.

### Fecal metabolomic profiling and water content quantification

Fecal samples were divided into homogeneous aliquots shortly after collection and stored at −80 °C for subsequent analyses. One of the aliquots was further aliquoted into two parts – one for fecal metabolomic analysis and another for water content quantification.

Fecal metabolomic profiling was performed using nuclear magnetic resonance (^1^H-NMR) spectroscopy, which targets 68 commonly measured water-soluble fecal metabolites (Supplementary Data 1; TMIC, Edmonton, Canada), as detailed in Supplementary Methods. Briefly, approximately 100 mg of fecal sample was used to extract 200 μl fecal water, which was mixed with 50 μl NMR buffer for spectral analysis.

Lyophilization was performed to quantify fecal water content. Aliquoted samples were weighted after transferred to pre-weighted Eppendorf tubes. Samples were then frozen before putting into the lyophilizer overnight. After lyophilization, the weight of the Eppendorf tube was recorded, and the weight of dry sample was calculated.

### Enteropathy marker assessment

A separate fecal aliquot was used to measure fecal enteropathy markers, myeloperoxidase (MPO), calprotectin and alpha-1-antitrypsin (AAT), as detailed in Supplementary Methods. Briefly, The Easy Stool Extraction Device was used to extract fecal content from 15 mg stool per the manufacturer’s instructions (ALPCO, Salem, NH). MPO and AAT were quantified by commercially available ELISA kits (ALPCO, Salem, NH), and calprotectin was quantified using the diagnostic chemiluminescence ELISA kit per the kit insert (ALPCO, Salem, NH). Serum samples were used for quantifying I-FABP using a commercial ELISA kit (Hycult Biotech, Uden, Netherlands) according to package instructions.

Dry weight concentrations were derived based on water content measured by lyophilization. To compare marker ranges with the literature, wet weight concentrations were also analyzed. Results were related to clinical cut-off concentrations used to diagnose or monitor disease activity in inflammatory bowel disease or celiac disease: calprotectin: 200 μg/g, MPO: 2000 ng/ml, AAT: 270 ug/g^28^, and I-FABP: 450 pg/ml^29,30^.

### Statistical analyses

Metabolites that had coefficient of variation <30% in quality control samples and were detected in at least 80% of samples in either group were retained for subsequent analysis. Data preprocessing and imputation for missingness were conducted as detailed in Supplementary Methods. Both univariate and multivariate approaches were used to reveal features and differences in patterns of analytes associated with mortality. First, to identify individual analytes associated with mortality, univariate conditional logistic regression accounting for the matched design was performed^31^. Potentially significant analytes were determined based on *P* < 0.05, which were obtained from two-sided Wald tests and were adjusted for multiple testing to control for false discovery rate (FDR) according to the Benjamini and Hochberg. Then, multivariable analysis by elastic net penalized logistic regression was implemented using the “glmnet” R package^32^, as detailed in Supplementary Methods. Analytes that with *P* < 0.05 in univariate analysis or influential (>80% bootstrap confidence interval (CI) of coefficient not crossing zero) in multivariable analysis were considered as differential analytes. The ‘mixOmics’ R package was used to analyze the differential analytes. To visualize the univariate association between levels of differential metabolites and the probability of being a case in the study sample, the histSpikeg function from the ‘Hmisc’ R package was used. For enteropathy markers, univariate conditional logistic regression was used for group comparisons, except for I-FABP. Due to serum sample availability constraints, among the case-control pairs, several pairs had only the case or the control samples measured for I-FABP. To study I-FABP in an adequate sample size (*n*_case_ = 57, *n*_control_ = 61) without dropping the unpaired samples, I-FABP was analyzed by logistic regression adjusted for the matching variables, age, MUAC and HIV. Sensitivity analyses were performed to account for potential confounders (edema, recruitment site, and breastfeeding status), as described in Supplementary Methods.

To understand the correlations between intestinal disturbances and systemic inflammation, we integrated the intestinal data in the precent study and systemic data from a previous study. In the previous study, we quantified systemic inflammatory marker and SCFA levels among children from the same cohort, using the Luminex assay (EMD Millipore, Burlington, USA) and the LC-MS/MS-based TMIC PRIME® assay (TMIC, Edmonton, Canada), respectively, as detailed in^33,34^. Cross-correlation analysis was performed amongst the analytes based on pairwise Pearson’s correlations, with significance level at P_FDR_ < 0.05. PLS path modeling was used to study the causal relationships among intestinal factors, systemic factors, and mortality, using the “plspm” R package^35^. Patients with complete data were included to this analysis. All manifested variables were log_10_-transformed and standardized before modelling. Path coefficients and their 95%CI were validated using bootstrap resampling.

### Ethical approval

Ethical approval for this study was obtained from the College of Medicine Research and Ethics Committee of the University of Malawi, the KEMRI Scientific Ethical Review Committee, Kenya, the Oxford Tropical Research Ethics Committee, and the Hospital for Sick Children, Toronto, Canada. The trial sponsor was the University of Oxford. Informed consent was obtained from parents or caregivers prior to the enrollment of all study participants.

### Reporting summary

Further information on research design is available in the Nature Portfolio Reporting Summary linked to this article.

## Results

### Characteristics of study patients

Table 1 is the characteristics of study participants at admission. Children included in this matched case-control study have a median age of 15 months (IQR: 10–26) and 47% are female. There is a higher proportion of children with nutritional edema among NS (43% vs. 24%, *p* = 0.02). The proportion of children with diarrhea does not differ between NS and S, similar to that in the parent trial (*p* > 0.05). Other characteristics are similar between NS and S. The median time to death for NS is 6 days (IQR: 4–10) and the time to discharge for S is 8 days (IQR: 7–11) since hospital admission.

**Table 1:** Admission characteristics of participants with fecal samples analyzed.

	Trial participants				Admission samples			
	NS (*n* = 127)	S (*n* = 653)	OR	[95% CI]	NS (*n* = 68)	S (*n* = 68)	OR	[95% CI]
**Anthropometric & Nutritional Features**								
Age (months)^a^, mean ± sd	21.4 ± 16.6	22.7 ± 18.7	0.9	[0.8, 1.1]	19.6 ± 13.4	19.5 ± 12.6	1	[0.7, 1.5]
Sex (female), n (%)	64 (50%)	296 (45%)	1.2	[0.8, 1.8]	32 (47.1%)	33 (48.5%)	0.9	[0.5, 1.8]
MUAC (cm)^a^, mean ± sd	10.7 ± 1.6	11.3 ± 1.4	0.6	[0.5, 0.8]	10.6 ± 1.5	10.8 ± 1.5	0.9	[0.6, 1.3]
WHZ, mean ± sd	−3.8 ± 1.5 (*n* = 120)	−3.2 ± 1.5 (*n* = 617)	0.7	[0.5, 0.8]	−3.9 ± 1.6 (*n* = 65)	−3.5 ± 1.6	0.8	[0.6, 1.1]
WAZ, mean ± sd	−4.3 ± 1.4	−3.9 ± 1.4 (*n* = 651)	0.6	[0.5, 0.8]	−4.4 ± 1.4	−4.2 ± 1.4	0.8	[0.5, 1.3]
HAZ, mean ± sd	−3.3 ± 1.7 (*n* = 124)	−3.0 ± 1.7 (*n* = 652)	0.8	[0.6, 1]	−3.2 ± 1.8 (*n* = 66)	−3.3 ± 1.5	1.1	[0.7, 1.7]
Nutritional oedema present, n (%)	47 (37%)	199 (31%, *n* = 650)	1.3	[0.9, 2]	29 (43%)	16 (24%)	2.4	[1.2, 5.1]
Breastfed, n (%)	53 (42%)	284 (44%)	0.9	[0.6, 1.4]	30 (44%)	34 (50%)	0.8	[0.4, 1.6]
**Clinical Features**								
HIV antibody test^a^, n (%)								
Negative	64 (50%)	507 (77%)		Ref	38 (56%)	38 (56%)		Ref
Positive	47 (37%)	122 (19%)	3.1	[2, 4.7]	23 (34%)	23 (34%)	1	[0.5, 2.1]
Refused/Died before testing	16 (13%)	24 (3.7%)	5.3	[2.7, 10.5]	7 (10%)	7 (10%)	1	[0.3, 3.1]
Diarrhoea, n (%)	61 (48.0%)	267 (41%)	1.3	[0.9, 2]	37 (54%)	27 (40%)	1.8	[0.9, 3.6]
Severe Pneumonia, n (%)	40 (32%)	153 (23%)	1.5	[1, 2.3]	24 (35%)	20 (29%)	1.3	[0.6, 2.7]
Chest indrawing	39 (31%)	105 (16%)	2.3	[1.5, 3.6]	23 (34%)	14 (21%)	2	[0.9, 4.3]
Fever, n (%)	36 (28%)	180 (28%)	1	[0.7, 1.6]	20 (29%)	18 (27%)	1.2	[0.5, 2.4]
Vomiting, n (%)	32 (25%)	183 (28%)	0.9	[0.6, 1.3]	15 (22%)	16 (24%)	0.9	[0.4, 2.1]
Impaired consciousness, n (%)	10 (7.9%)	18 (2.8%)	3	[1.4, 6.7]	5 (7.4%)	4 (5.9%)	1.3	[0.3, 4.9]
Cerebral palsy, n (%)	17 (13%)	99 (15%)	0.9	[0.5, 1.5]	11 (16%)	14 (21%)	0.7	[0.3, 1.8]
Chronic cough, n (%)	6 (4.7%)	44 (6.7%)	0.7	[0.3, 1.6]	3 (4.4%)	7 (10%)	0.4	[0.1, 1.6]
Hypothermia, n (%)	8 (6.3%)	35 (5.4%)	1.2	[0.5, 2.6]	6 (8.8%)	6 (8.8%)		NA
Convulsions, n (%)	7 (5.5%)	30 (4.6%)	1.2	[0.5, 2.8]	3 (4.4%)	1 (1.5%)	3.1	[0.3, 30.5]
Malaria, n (%)	6 (4.7%)	57 (8.7%)	0.5	[0.2, 1.2]	3 (4.4%)	5 (7.4%)	0.6	[0.1, 2.5]
Tuberculosis, n (%)	4 (3.1%)	12 (1.8%)	1.7	[0.6, 5.5]	1 (1.5%)	3 (4.4%)	0.3	[0, 3.2]
Anemia, n (%)	4 (3.1%)	22 (3.4%)	0.9	[0.3, 2.8]	3 (4.4%)	0 (0.0%)		NA
**Original Trial Features**								
Intervention arm, n (%)	68 (53.5%)	322 (49.3%)	1.2	[0.8, 1.7]	38 (55.9%)	26 (38.2%)	2	[1, 4.1]
Recruitment site, n (%)								
Queen Elizabeth Central Hospital	64 (50%)	247 (38%)		Ref	35 (52%)	27 (40%)		Ref
Kilifi County Hospital	23 (18%)	156 (24%)	0.6	[0.3, 1]	8 (12%)	12 (18%)	0.5	[0.2, 1.4]
Coast Provincial General Hospital	40 (32%)	250 (38%)	0.6	[0.4, 1]	25 (37%)	29 (43%)	0.7	[0.3, 1.4]

^a^Factors used in propensity score matching of case-controls. *n* number of study participants, *OR* odds ratio, *CI* confidence interval, *MUAC* mid-upper arm circumference, *WHZ* weight for height z score, *WAZ* weight for age z score, *HAZ* height for age z score, *sd* standard deviation, *Ref* reference level (OR = 1). Note: ORs describe differences between S and NS groups rather than a statistical analysis of factors associated with death.

### Fecal SCFAs are not associated with mortality in children with CSM

**Fig. 1:**
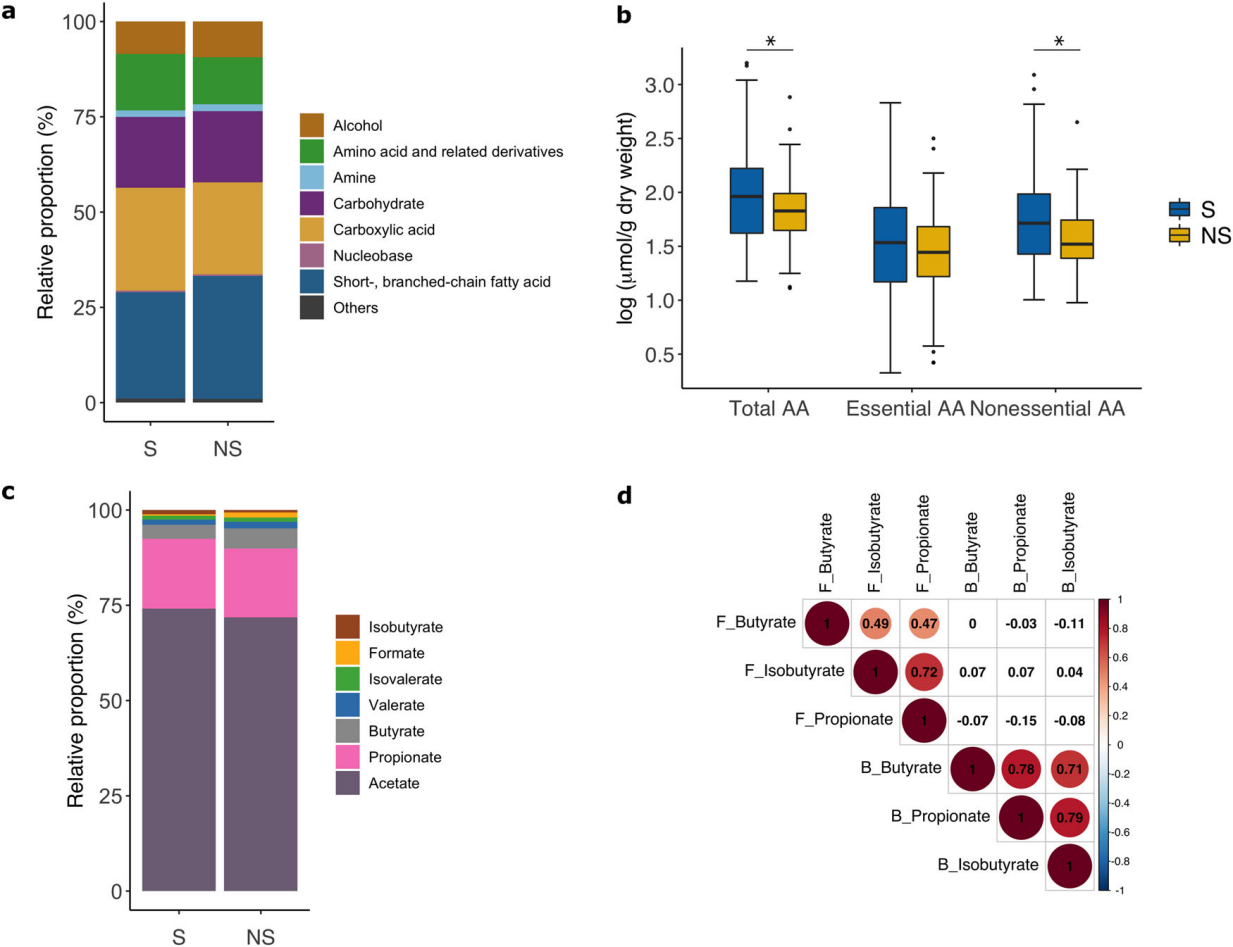
Fecal metabolome composition. **a** Relative proportion of metabolites in each class. The class Others includes metabolites presence in relatively small amounts of the classes of phenol, phenethylamine, phenylpropanoid, sulfonic acid or carbonyl compound. **b** Boxplots (center line: median; box limits: IQR; whiskers: 1.5 times IQR) showing concentrations of amino acids between survivors (S) and nonsurvivors (NS). **c** Relative proportions of individual SCFA species to total SCFAs. (**d**) Pairwise Pearson correlations between fecal (labeled as “F_”) and circulating (labeled as “B_”) SCFAs. Significant (*P*_FDR_ < 0.05) correlations are denoted by color-filled circles. Unless stated otherwise, *n* = 68 cases and 68 controls.

Of 68 metabolites targeted, 61 fulfill the criteria for measurement quality and are retained for further analysis (Supplementary Data 1). These metabolites are grouped into seven classes with SCFA (30%), and carboxylic acids (26%), followed by carbohydrates (19%), amino acids and derivatives (13%), and alcohols (9.0%) being the dominant metabolite classes constituting the fecal metabolome of the study population (Fig. 1a). Between NS and S, total metabolite abundance, relative proportion, or mean absolute concentration of individual metabolite classes do not differ, except for the class of amino acids. At admission, mean concentration of total amino acids and derivatives are lower in NS than S (95.8 ± 107.5 vs. 191.8 ± 314 μmol/g dry weight, OR [95%CI] = 0.39 [0.16, 0.96], *P*_raw_ = 0.04, *P*_edema adjusted_ = 0.01, Fig. 1b). This difference is driven by reductions in non-essential amino acids (alanine, aspartate, glutamate, glutamine, glycine, proline, serine, and tyrosine) among NS.

SCFAs are the main fermentation products of the gut microbiota playing an important role in intestinal homeostasis. Acetate followed by propionate and butyrate are the most abundant species among the SCFAs, consistent with their relative proportion in well-nourished populations^36^. Between NS and S, total SCFAs and individual SCFA species do not differ in their relative proportions nor absolute concentrations (Fig. 1c).

Using serum data of propionate, butyrate and isobutyrate, we examine if levels of these SCFA species in the feces are correlated with systemic levels. We found that although correlations within the same biofluid are strong, fecal SCFA levels do not correlate with circulating levels (Fig. 1d).

### Fecal metabolic signatures are associated with mortality

**Fig. 2:**
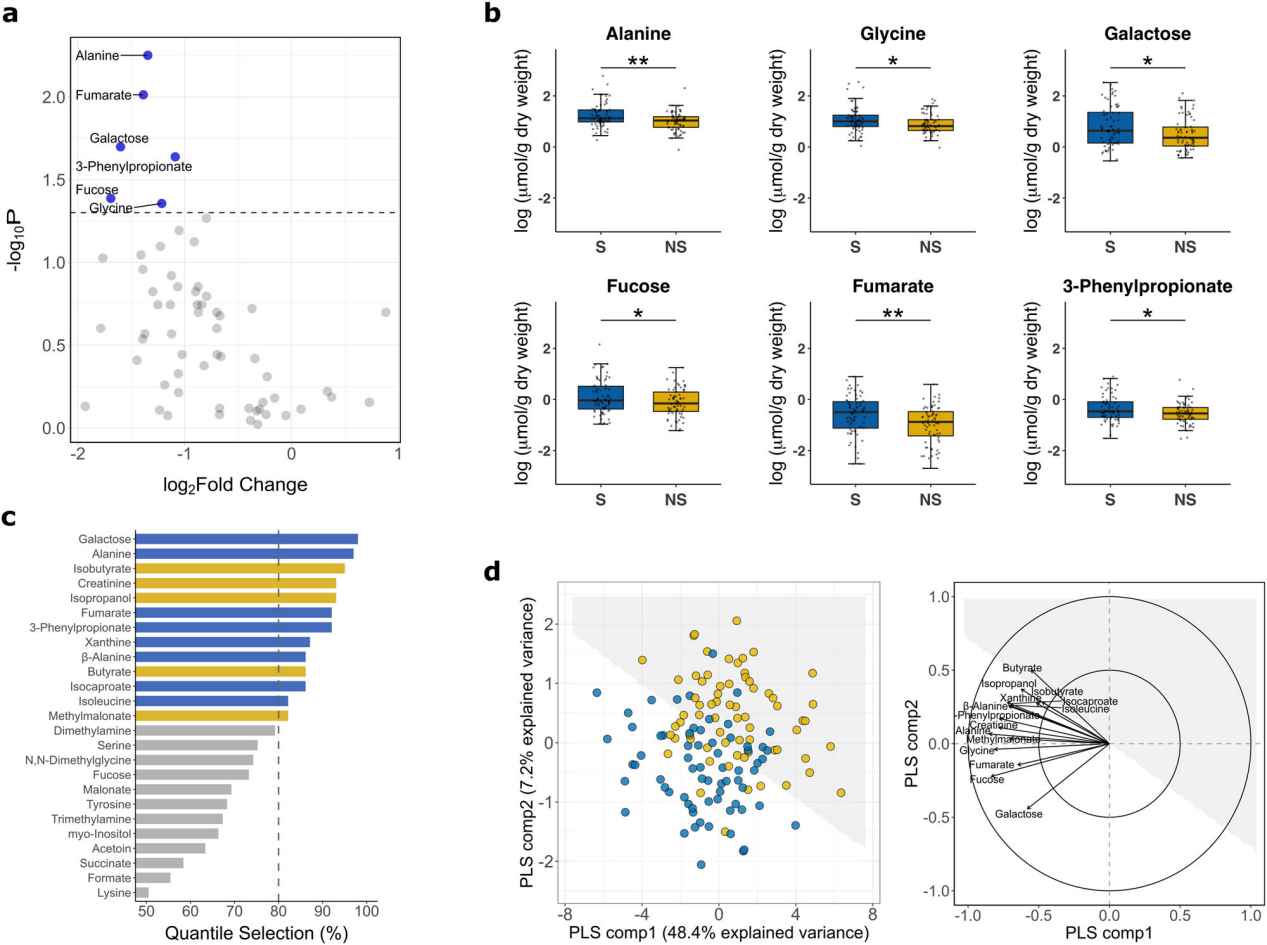
Fecal metabolomic signatures associated with mortality. **a** Volcano plot of 61 measurable metabolites. Univariately differential metabolites between nonsurvivors (NS) and survivors (S) are above the dashed line (*P* = 0.05). **b** Boxplots (center line: median; box limits: IQR; whiskers: 1.5 times IQR) showing concentrations of univariately differential metabolites between NS and S. *P < 0.05, ** *P* < 0.01. **c** Multivariable elastic net analysis with quantile selection. Bars correspond to the bootstrapped confidence interval of a respective metabolite being selected (i.e. coefficient ≠ 0) by the elastic net model; influential metabolites are those above the threshold of 80% (dashed line). Bar color denotes median concentration higher (yellow) or lower (blue) in NS compared to S. **d** Partial least squares discriminant analysis (PLS-DA) of the combined profile of 15 differential features. Left: score plot of individuals (NS in yellow; S in blue) clustered by multilevel PLS-DA. The interface between the white and the grey shaded area represents the classification decision line. Right: correlations among the differential features with PLS components. Arrows denote the direction and magnitude of correlations. Model performance and validity measures were cross-validated AUC = 0.72 ± 0.03, misclassification rate=0.35 ± 0.03. Unless stated otherwise, *n* = 68 cases and 68 controls.

Comparing the fecal metabolome of NS to S, 6 metabolites are univariately lower in NS (*P*_raw_ < 0.05), including 2 amino acids (alanine and glycine), 2 carboxylic acids (fumarate and 3-phenylpropionate) and 2 carbohydrates (galactose and fucose) (Fig. 2a, b). However, these metabolites do not reach significance after multiple testing correction (*P*_FDR_ > 0.05). It can also be seen from the unsymmetrical volcano plot that the majority of the fecal metabolites are lower in NS compared to S. The association between individual metabolite and mortality is shown in Supplementary Fig. 2 and Supplementary Data 2. For example, for an increase from 25^th^ to 75^th^ percentile in alanine concentration (from 7.5 to 23 μmol/g), the associated odds of death decreases by a factor of 0.52 (95% CI: 0.33–0.82).

In multivariable analysis, 13 metabolites are identified as influential features in distinguishing NS from S (Fig. 2c), including alanine, fumarate, 3-phenylpropionate, and galactose that are also identified as univariately differential, and 9 additional influential metabolites (xanthine, β-alanine, isocaproate, isoleucine, creatinine, isobutyrate, isopropanol, butyrate, methylamalonate). Supplementary Fig. 3a is a Venn diagram showing the overlap between univariate and multivariable analyses. For each matched case-control pair, the combined profile of the univariate and multivariable differential features (total 15 metabolites) only moderately discriminate NS from S (AUC = 0.72). As illustrated in Fig. 2d, differential features most strongly driving the separation between NS and S cluster (i.e. arrows perpendicular to the decision line) are reductions of galactose, fucose, and fumarate in NS. As the concentration of these features increase, the observed probability of death within the case-control sample set decreases steadily (Supplementary Fig. 3b). Conversely, features that are parallel to the decision line, particularly butyrate, isobutyrate, isocaproate and xanthine, show a more complex relationship with the observed probability of death (Supplementary Fig. 3c). Further adjusting the data by edema, site, or breastfeeding status do not fundamentally change the results (Supplementary Fig. 4).

### Enteropathy is present in CSM but is not associated with mortality

**Fig. 3:**
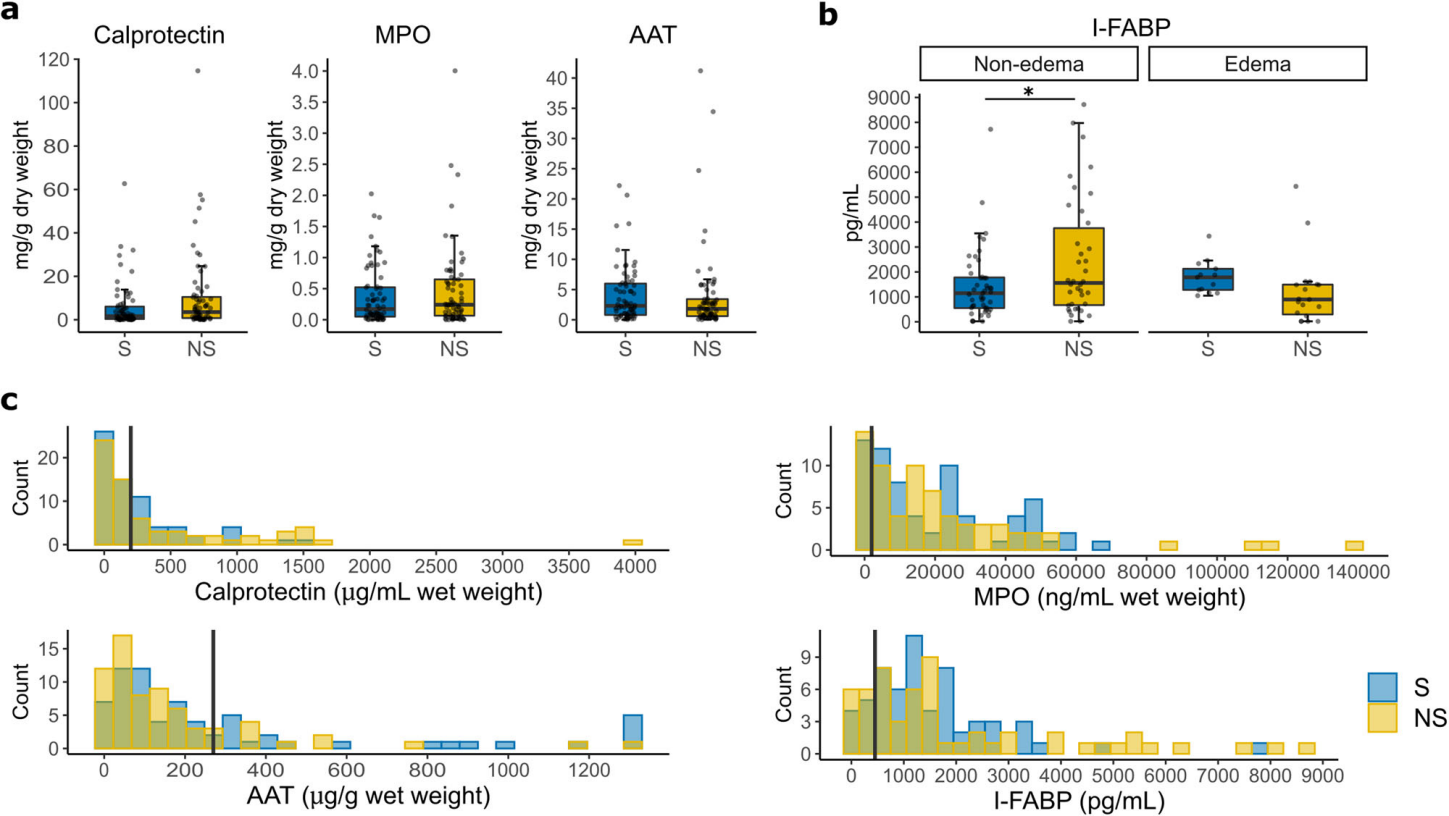
Enteropathy marker levels at admission between survivors and nonsurvivors. **a** Concentrations of fecal enteropathy markers in dry fecal weight between nonsurvivors (NS) and survivors (S): Calprotectin (P = 0.08), Myeloperoxidase (MPO, *P* = 0.27), Alpha-1 antitrypsin (AAT, *P* = 0.54). (**b**) Serum marker Intestinal fatty acid binding protein (I-FABP) concentrations stratified by edema status between NS and S (*n* = 57 cases and 61 controls with one outlier in controls removed; *P*_non-edema_ = 0.029, *P*_edema_ = 0.91, P_interaction_ = 0.082). **c** Histograms showing distribution of individual enteropathy marker between NS and S. Concentrations are expressed in wet fecal weight. Vertical line indicates clinical cutoffs for normal marker levels in non-tropical settings (calprotectin: 200 μg/g wet feces, MPO: 2000 ng/ml wet feces, AAT: 270 ug/g wet feces, I-FABP: 450 pg/ml), marker levels above these cutoffs are considered elevated^28–30^. Unless stated otherwise, *n* = 68 cases and 68 controls. Each boxplot shows the median (center line), IQR (box limits), and data points with whiskers showing 1.5 times IQR. * *P* < 0.05.

To evaluate associations between enteropathy and mortality, admission levels of one serum and three fecal markers for intestinal function are compared between NS and S. We found that levels of calprotectin, MPO, or AAT do not differ between NS and S, with or without adjusting for edema or site (*P*_raw_ > 0.05, *P*_adjusted_ > 0.05) (Fig. 3a). The serum marker I-FABP do not differ between NS and S. Upon sensitivity analysis examining effects of nutritional edema, weak evidence for an interaction between I-FABP and edema status is revealed (*P*_interaction_<0.1). Specifically, among children without edema, I-FABP levels are elevated (OR [95%CI] = 1.0003 [1.0000, 1.0006], *P* = 0.029) in NS, while among children with edema, I-FABP levels do not differ between groups (Fig. 3b). Notably, I-FABP has a weak association, while the fecal markers have no or inconsistent associations with diarrhea at admission and within 48 h postadmission (Supplementary Fig. 5).

Fecal marker levels and their clinical reference ranges are often expressed as wet weight concentrations in the literature. To compare fecal marker levels of our study population to these reference ranges, wet weight concentrations are also examined (Fig. 3c). A considerable proportion of patients has elevated levels of calprotectin, MPO and AAT based on reported references ranges (calprotectin: > 200 μg/g wet feces, MPO: > 2000 ng/ml wet feces, AAT: > 270 ug/g wet feces)^28^, but the proportion of patients with elevated levels do not significantly differ between NS and S (calprotectin: 43% vs 40% elevated; MPO: 81% vs 84% elevated; AAT: 18% vs 31%). Similarly, a considerable proportion of patients have elevated levels of serum I-FABP based on a cut-off used for detecting celiac disease in well-nourished populations (I-FABP: > 450 pg/ml^29,30^), but the proportions do not differ between NS and S (79% vs 85%). These data suggest that signs of enteropathy are present in children with CSM upon admission, but the degree of enteropathy is not associated with mortality.

### Interrelationships between intestinal disturbances, systemic inflammation, and mortality

Cross-correlation analysis between enteropathy markers, fecal SCFAs, and systemic markers analytes is conducted. The two fecal markers for intestinal inflammation, calprotectin and MPO, are strongly correlated with each other (r_calprotectin~MPO_ = 0.8, *P*_FDR_ < 0.0001). They are moderately correlated with AAT (r_MPO~AAT_ = 0.35, r_calprotectin~AAT_ = 0.3, *P*_FDR_ < 0.001). Interestingly, calprotectin is also positively correlated with circulating SCFA levels (r_calprotectin~SCFA_ = 0.3, P_FDR_ = 0.001) but not with fecal SCFAs. This indicates a link between intestinal inflammation and systemic levels of SCFAs, which are metabolic products originated from the gut microbiota.

**Fig. 4:**
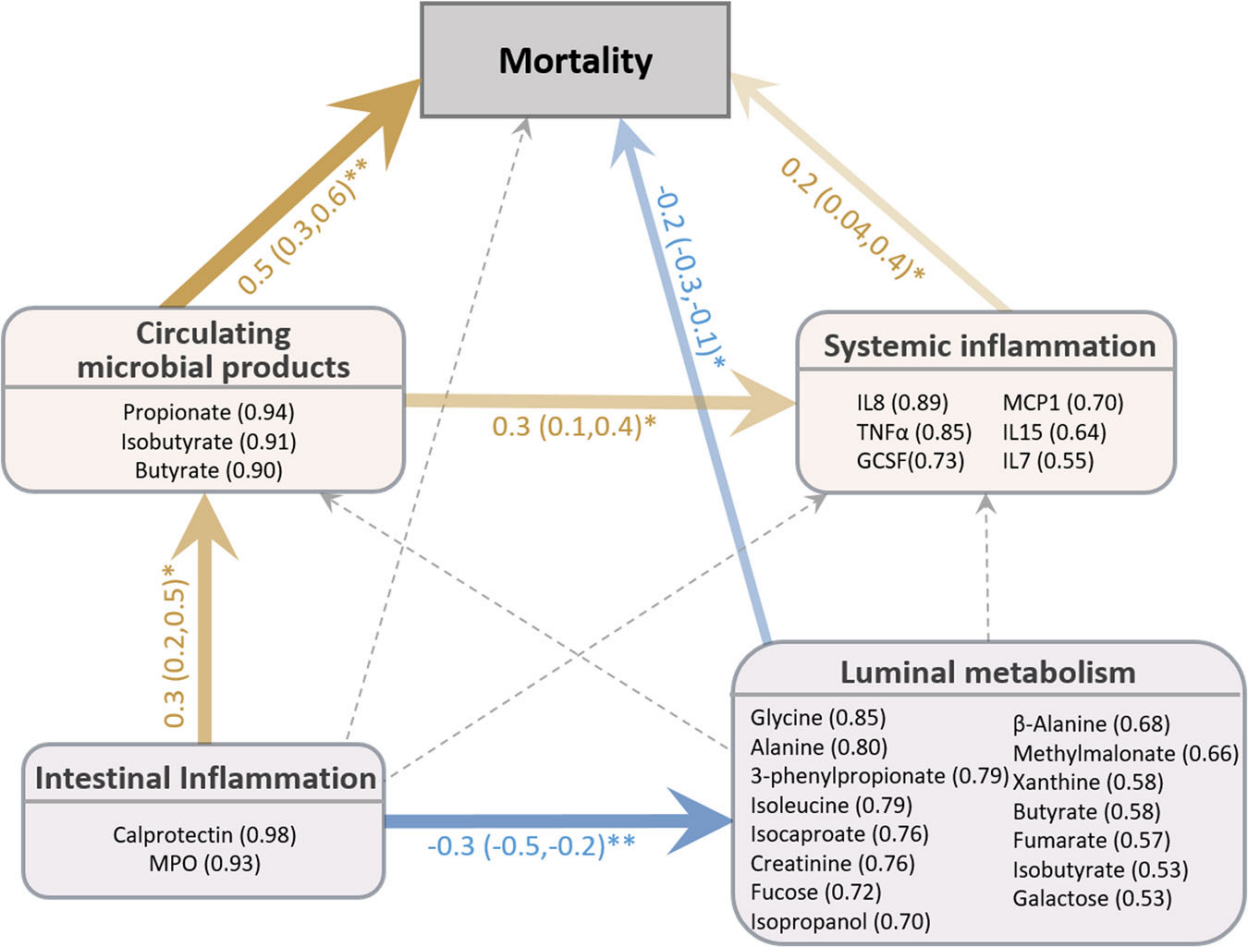
Relation between intestinal disturbances, systemic inflammation, energy metabolism and mortality estimated by PLS path modeling. Children with both blood and fecal data measured were included in this analysis (*n* = 51 cases and 42 controls). Latent variables are represented by rounded rectangles. Their corresponding observed manifested indicators and respective loadings are listed underneath. Model goodness of fit = 0.3, R^2^ = 0.4. Path coefficients above each interconnecting arrow indicate the strength and direction of the relation between nodes of the model. Bootstrap resampling was used to validate significance of path coefficients and 95%CI are presented next to the coefficients. Significant paths are marked by solid arrows (positive and negative associations are denoted by yellow and blue arrows, respectively); tested but insignificant paths are indicated by dashed arrows in light grey. *P < 0.05, ** *P* < 0.01.

To further decipher the interrelationships among intestinal disturbances, systemic inflammation, systemic microbial product levels, and mortality, PLS path modeling is conducted. Specifically, 4 latent variables are constructed from manifested indicators: 1) Luminal metabolism (indicators: 15 differential fecal metabolites), 2) Intestinal inflammation (indicators: calprotectin and MPO), 3) Systemic inflammation (indicators: proinflammatory mediators IL7, IL8, IL15, TNFa, GCSF, MCP1), and 4) Circulating microbial products (indicators: serum propionate, butyrate, and isobutyrate), as illustrated in Fig. 4. Communality within each latent variable is high, suggesting that the latent variables summarize the variability of the indicators well, satisfying model specification requirements. Strengths and directions of the interrelationships are denoted by arrows in Fig. 4. Consistent with results in the above sections, intestinal inflammation, marked by calprotectin and MPO, do not directly correlate with mortality (direct effect = −0.05 (95%CI: −0.2, −0.08)). However, the model shows significant indirect effects on mortality via a positive association with circulating microbial products leading to systemic inflammation, as well as a negative association with luminal metabolic profiles (total indirect effect = 0.23 (95%CI: 0.12–0.33)). Although intestinal inflammation is not directly linked to mortality, intestinal inflammation contributed to mortality indirectly via reducing luminal metabolism and increasing the systemic load of microbial products, thus indirectly contributing to mortality. We also examined the role of diarrhea in these causal pathways. A history of diarrhea at admission is not associated with any path nor improves model fit, and thus diarrhea is not included to the final model.

## Discussion

This case-control study, nested within a clinical trial, compares the fecal metabolome and a set of enteropathy markers between survivors and nonsurvivors of hospitalized children with severe malnutrition. We assess the association between intestinal disturbances and mortality among severely malnourished children by examining multiple enteropathy markers and the fecal metabolome. At admission, the fecal metabolome of nonsurvivors is characterized by reductions in certain amino acids, monosaccharides, and other microbial fermentation products. In contrast to the initial speculation, fecal SCFAs do not differ between groups. A considerable proportion of children in the study has elevated enteropathy marker levels. Overall, enteropathy markers are not directly associated with mortality, but an indirect association between intestinal inflammation and mortality is observed. The presence of diarrhea at admission is not associated with mortality and has limited or no association with enteropathy markers. These findings suggest a limited contributory role of enteropathy in CSM mortality.

The differences in fecal metabolome between nonsurvivors and survivors are driven by reductions of galactose, fucose, alanine, glycine, fumarate and 3-hydroxyphenylpropionate. Galactose is a monosaccharide produced from lactose in milk by digestive enzymes or microbial fermentation. Lower fecal galactose in nonsurvivors compared to survivors may reflect reduced recent dietary intake of carbohydrates. Fucose can be liberated from fucosylated glycans by certain bacteria, such as *Bifidobacterium*, *Bacteroides* and mucin degraders; and it can be utilized as energy substrates by bacteria^37^. Reduced fecal levels of fucose among nonsurvivors may be attributed to reduced intestinal abundance of fucosylated glycans, reduced fucose producers or increased fucose utilizers in the gut microbiota. Notably, fucosylation of the intestinal epithelium are necessary for establishing the host-commensal symbiotic relationships and colonization resistance against pathogens. Deficient of functional expression of the fucosylation enzyme in both mice and humans are associated with increased susceptibility to various intestinal infections and inflammatory diseases, including Crohn’s disease^38^. Fumarate is a fermentation intermediate and 3-hydroxyphenylpropionate is a major fermentation end product of aromatic amino acids^11^.

Total amino acids is lower in nonsurvivors than survivors. In general, reductions in these fecal metabolites may reflect compositional and functional differences of the gut microbiota. It is plausible that, instead of contributing to subsequent mortality, the reduction in fecal metabolites is attributed to illness-induced anorexia or microbiome perturbations due to preadmission antibiotic exposures. This may indicate that these children were sicker at admission. However, without microbiota, intestinal absorption, and pre-admission medication data, this remains speculative.

SCFAs do not differ between nonsurvivors and survivors. This contrasts with previous data that showed reductions in fecal butyrate and propionate at admission among children who died in hospital^25^. However, the reported significant associations may be biased by the small sample size (*n*_recovery_ = 52, *n*_death_ = 9) of the previous study, considering the known high variability of human fecal SCFAs^39,40^. The lack of association with mortality revealed in the present study suggests that the abundances of SCFA-producing bacteria in the gut microbiota may be similar between survivors and nonsurvivors. Despite that SCFAs, particularly butyrate, are known to have anti-inflammatory and barrier-enhancing effects to the intestine^41,42^, their roles in mortality are unclear. A recent study showed that although fecal SCFA levels were lower in patients with sepsis, they are not associated with mortality^43^. Clearly, fecal SCFA levels are influenced by many factors, such as intake, absorption, utilization by enterocytes, transit time and antibiotic use, which are not assessed in our study. As a matter of fact, we found no correlation between fecal and circulating SCFA levels.

A set of markers are evaluated covering different domains of enteropathy, including intestinal inflammation, permeability and damage. We found that a substantial proportion of children has elevated marker levels when compared to reference ranges used for nourished population. This is consistent with the high prevalence of enteropathy seen in low-resource settings. For example, a birth cohort of 700 infants living in urban slum in Bangladesh reported that over 80% of infants had elevated levels of calprotectin, MPO and AAT, when applying clinically used references. It’s worth noting that there are no clear clinical cut-offs of enteropathy markers established for malnourished children^44^.

Levels of fecal enteropathy markers are not statistically different between nonsurvivors and survivors, but a trend towards increased calprotectin was seen in nonsurvivors (*p* < 0.1). Calprotectin and MPO are proteins released by neutrophils and their fecal levels indicate mucosal neutrophil activity and inflammation. Aligned with their common roles, calprotectin and MPO levels are strongly correlated. AAT is a circulating protein synthesized by the liver and increased fecal excretion of AAT is an indicator of gut leakiness. AAT production is affected by hepatic synthetic activity, which is impaired in malnutrition^45^. A more comprehensive test, such as AAT clearance calculated from 24-hour blood and stool collection, may be required for reliable use of AAT to assess of gut permeability^46^. I-FABP is uniquely expressed at the tips of villi of differentiated enterocytes, and it is only released into systemic circulation upon enterocyte deaths during inflammation or injury^47^. Elevated circulating I-FABP is an indicator of villus atrophy and epithelial damages. I-FABP is the only enteropathy marker in our study that has a weak association with the presence of diarrhea at admission and 48-hour postadmission, which may reflect its physiological relevance with diarrhea compared to the other markers. In non-edematous patients, admission I-FABP levels are significantly higher in nonsurvivors than survivors, but no difference is found for edematous patients. Further investigation is warranted to understand if the accumulation of fluid within the interstitial spaces in edema would affect serum I-FABP levels or if physiological differences between malnutrition phenotypes^48^ would have impacts on I-FABP expression in the intestine.

To reconcile how and to what extent these intestinal factors contribute to mortality, admission intestinal, systemic and clinical data are integratively analyzed using the path analysis. We found that intestinal inflammation is not directly associated with systemic inflammation nor mortality, but indirectly via increasing systemic microbial products as marked by circulating SCFAs. In a large multi-country community cohort, using similar causal path modeling methods, Kosek et al. found conflicting relationships between intestinal inflammation markers (MPO, neopterin) and systemic inflammation, although intestinal inflammation and systemic inflammation are both positively associated with enteric pathogen exposure^49^. We also found that increased intestinal inflammation negatively influenced luminal metabolite abundances. It has been reported in patients with inflammatory bowel disease that fecal metabolites and metabolite classes are frequently depleted^50^.

The study has several limitations. First, fecal material is a complex matrix constituting an array of inputs from host, microbiota, and diet. It is not unusual to see high variability in fecal data as a result of many confounding factors such as diet, age, bowel activity, and water content^51^. Human feces are known to contain about 60–85% water and such a wide range of variation can have a significant impact on statistical inference^51^. To control for some inherent variability, we account for sample water content in the quantification of fecal analytes, which is currently not commonly done in majority of the fecal studies. Nonetheless, this study only examines factors associated with mortality at a single time point on admission and there could have confounding factors that are not measured or accounted for. For example, without pre-admission data, it is plausible that the findings on reduced fecal metabolite abundances in nonsurvivors are confounded by antibiotic use prior to admission. Similarly, factors post-admission, such as new onset of infections during hospitalization, could also influence the intestinal environment and mortality risk. Future work should consider integrating microbiome, pre-admission medication and dietary data with serial fecal metabolomics to better disentangle the interrelationships. Second, despite a relatively large and balanced sample size compared to the previous study on enteropathy and mortality in CSM^25^, it may still be insufficient to overcome the inherent variability of fecal analytes, which can reduce statistical power to detect differences. For this reason, a relaxed statistical significance criterion of *p* < 0.05 is used in fecal metabolomic analysis with p-values corrected for multiple comparisons found in Supplementary Data 2. While the chosen cutoff may be considered liberal, a more stringent cutoff could restrict our ability to identify important metabolites with real effects, particularly given the known high variability of fecal analytes. Therefore, the cutoff is chosen to balance the trade-off between false positives and false negatives. Similarly, when determining influential features from bootstrapped samples, 80% is chosen as a cutoff instead of a higher probability (i.e., 95%) in order to reduce the risk of missing true effects. It is worth noting that performing the bootstrap validation step on elastic-net regression, instead of taking results directly from one elastic-net regression, is a procedure implemented to protect against false positives. Nonetheless, we acknowledge that the significant associations reported in our study may be still influenced by the sample size, considering the high variability inherent to human fecal analytes. Consequently, our findings should be interpreted with caution and warrant further validation through larger-scale studies to robustly establish the relationship between intestinal disturbances and mortality in CSM. Third, as SCFAs are not specific markers for microbial translocation, quantifying other indicators of microbial translocation, such as LPS binding protein, endotoxin core antibody or sCD14^52^, could be helpful in supporting the associations that we observed. Notably, there is currently no consensus on a sensitive and reliable marker for microbial translocation. Lastly, the matched case-control design provides efficiency but also inevitably entails limitations. Our study aims to access the associations between intestinal features at admission and mortality among children with similar underlying mortality risks by controlling for potential confounding effects of age, wasting, and HIV. It is plausible that intestinal disturbances may have differential effects between different levels of the matched variables; for example, increased intestinal permeability may have a greater impact on HIV-positive than negative patients. However, effects of the matched variables cannot be evaluated within a matched the design. Therefore, our findings should be interpreted in the context of the design and population, and may not be generalizable to other conditions. The case-control design does not take into account the temporal relationship between exposure and outcome. A case-cohort design could be considered for future work aiming to study associations between intestinal features and time-to-death.

In conclusion, in children with CSM, signs of enteropathy appeared indirectly related to mortality via systemic inflammation, suggesting that future interventions should aim at both reducing systemic inflammation and intestinal inflammation, potentially by modulating the gut microbiome and barrier function, in order to improve clinical outcomes in this vulnerable patient population.

### Supplementary information

Supplementary Information

Description of Supplementary Materials

Supplementary Data 1

Supplementary Data 2

Supplementary Data 3

Reporting Summary

## Data Availability

All data supporting the findings of the study are in the main text, Supplementary Information, and public data repository. Supplementary Data 3 contains source data for the main figures in this manuscript. The fecal metabolomics and enteropathy marker data were deposited into the KEMRI-Wellcome data repository on the Harvard Dataverse under 10.7910/DVN/I4EYDR^53^. The clinical data of the parent trial and the systemic data are accessible from the same repository under 10.7910/DVN/N4RISX^54^ and 10.7910/DVN/GI8YL9^34^. All other data are available from the corresponding author on reasonable request.
