# Supplementary material for: Intestinal disturbances associated with mortality of children with complicated severe malnutrition
Source: Commun Med (Lond). 2023 Sep 29;3:128. doi: 10.1038/s43856-023-00355-0 (PMC10541881; doi:10.1038/s43856-023-00355-0)
Supplement: Supplementary file 1 — Supplementary Information [file 43856_2023_355_MOESM1_ESM.pdf]

Intestinal Disturbances Associated with Mortality of Children with Complicated Severe Malnutrition

Bijun Wen [1,2], Amber Farooqui [2], Celine Bourdon [2,3], Nawar Tarafdar [2], Moses Ngari [3,4], Emmanuel Chimwezi [3], Johnstone Thitiri [3,4], Laura Mwalekwa [4,5], Judd L Walson[3,6], Wieger Voskuijl [3,7,8] James A Berkley [3,4,9], Robert HJ Bandsma [1,2,3,10*]

[1] Department of Nutritional Sciences, Faculty of Medicine, University of Toronto, Toronto, Canada.

[2] Department of Translational medicine, Hospital for Sick Children, Toronto, Canada.

[3] The Childhood Acute Illness & Nutrition Network, Nairobi, Kenya.

[4] KEMRI/Wellcome Trust Research Programme, Kilifi, Kenya.

[5] Department of Paediatrics, Coast General Hospital, Mombasa, Kenya.

[6] Departments of Global Health, Medicine, Pediatrics and Epidemiology, University of Washington, Seattle, USA

[7] Amsterdam Institute for Global Health and Development, Department of Global Health, Amsterdam University Medical Centres, Amsterdam, The Netherlands.

[8] Department of Paediatrics and Child Health, Kamuzu University of Health Sciences (formerly College of Medicine), Blantyre, Malawi.

[9] Centre for Tropical Medicine & Global Health, Nuffield Department of Medicine, University of Oxford, Oxford, United Kingdom.

[10] Department of Biomedical Sciences, Kamuzu University of Health Sciences (formerly College of Medicine), Blantyre, Malawi.

* Corresponding author: Robert Bandsma

Address: Department of Paediatrics and Nutritional Sciences, University of Toronto

555 University Avenue, Toronto, Ontario, Canada. M5G 1X8

Email: robert.bandsma@sickkids.ca

Supplementary Information

Supplementary Methods:

Fecal metabolomic profiling and water content quantification

Fecal metabolomic profiling was performed using targeted ^{1}H-NMR spectroscopy (TMIC, Edmonton, Canada https://www.metabolomicscentre.ca/new_service/36). Approximately 100 mg of fecal sample was homogenized and mixed with 500 ml of ice-cold water by vigorous vortexing and sonication at 4°C, followed by centrifugation to extract the fecal water (the clear supernatants). 200 µl of the resultant fecal water was mixed with 50 µl NMR buffer and then transferred into 3 mm NMR tube for spectral analysis. All ^{1}H-NMR spectra were collected on a 700 MHz Avance III spectrometer equipped with a 5 mm HCN Z-gradient pulsed-field gradient cryoprobe. ^{1}H-NMR spectra were acquired at 25°C using the first transient of the NOESY pre-saturation pulse sequence, chosen for its high degree of quantitative accuracy. Before spectral analysis, all free induction decays were zero-filled to 250K data points. The singlet produced by the DSS methyl groups was used as an internal standard for chemical shift referencing (set to 0 ppm) and quantification. All ^{1}H-NMR spectra were processed and analyzed using the Chenomx NMR Suite Professional software package version 8.1 (Chenomx Inc., Edmonton, AB). Most of the visible peaks were annotated with a compound name.

Enteropathy marker assessment

The Easy Stool Extraction Device with 1.5 ml prefilled universal extraction buffer was used to obtain 15 mg stool and extract fecal content in 1:100 dilution per the manufacturer's instructions (ALPCO, Salem, NH). The resultant extracts were stored at -20°C before subsequent quantification of calprotectin and AAT. For MPO, an unfilled Easy Stool Extraction Device was used for extraction in 0.75 ml MPO-specific buffer to yield 1:50 dilution from 15 mg stool, and extracts were used immediately for MPO quantification per manufacturer's instructions (ALPCO, Salem, NH). MPO and AAT were quantified by commercially available enzyme-linked immunosorbent assays (ELISA), and calprotectin was quantified using the diagnostic chemiluminescence ELISA kit per the kit insert (ALPCO, Salem, NH), with the final dilutions at 1:500, 1:12500 and 1:12500, respectively. Serum samples were used for quantifying I-FABP using a commercial ELISA kit (Hycult Biotech, Uden, Netherlands) according to package instructions at a

final dilution of 1:10. Samples were randomized across plates for each marker. Four-parameter sigmoidal standard curves were used to quantify marker concentrations using the “drc” R package [1]. Samples above the highest standard were further diluted as appropriate according to remaining sample volume and reagent availability. Samples below detection limit were replaced with half of the lowest limit of detection (LOD).

Data preprocessing and analysis of differential analytes

Among the retained metabolites, influential observations were inspected and removed based on PCA and hierarchical clustering with single linkage analyses. Non-detected values and missing data were inspected and variables with <10% missingness were imputed by bagged trees imputation from the “Caret” R package [2]. Prior to analysis, data was $\log_{10}$-transformed, scaled to unit variance by autoscaling and mean-centred.

Considering that both univariate and multivariable analyses could be relevant in understanding biological pathways [3], univariate and multivariable analyses were conducted to identify differential analytes. Conditional logistic regression was applied for univariate analysis to identified significant analytes. Elastic net penalized logistic regression was used for multivariable analysis to identify influential analytes. Elastic net penalized logistic regression penalizes a mix of the sum of squared coefficients and the sum of absolute coefficients to prevent model over-fitting. As some of the coefficients can be shrunken to zero, elastic net regularization removes uninformative and selects discriminant features. Bootstrap resampling was used to evaluate the robustness of selected analytes. Metabolites with 80% of their bootstrapped coefficient confidence interval not crossing zero were considered as influential features. Tuning of λ was done based on five-fold cross-validated misclassification error within each bootstrap sample, as previously described [4]. Using the “mixOmics” R package, the identified differential analytes were then included to a multilevel PLS-DA analysis to better visualize their interrelationships while accounting for the matching design [5,6]. Ten-fold cross-validation was used to assess discriminant performance [7].

Sensitivity analyses

To examine the robustness of the study findings the following sensitivity analyses were conducted: 1) the main analyses were additionally adjusted for admission edema status, considering the slight imbalance of edema prevalence between case and control; additionally, interaction between edema and individual enteropathy marker was tested at $P_{interaction}<0.1$; 2) given that diet and treatments before hospitalization could be different between sites, the main analyses were additionally adjusted for sites and breastfeeding status

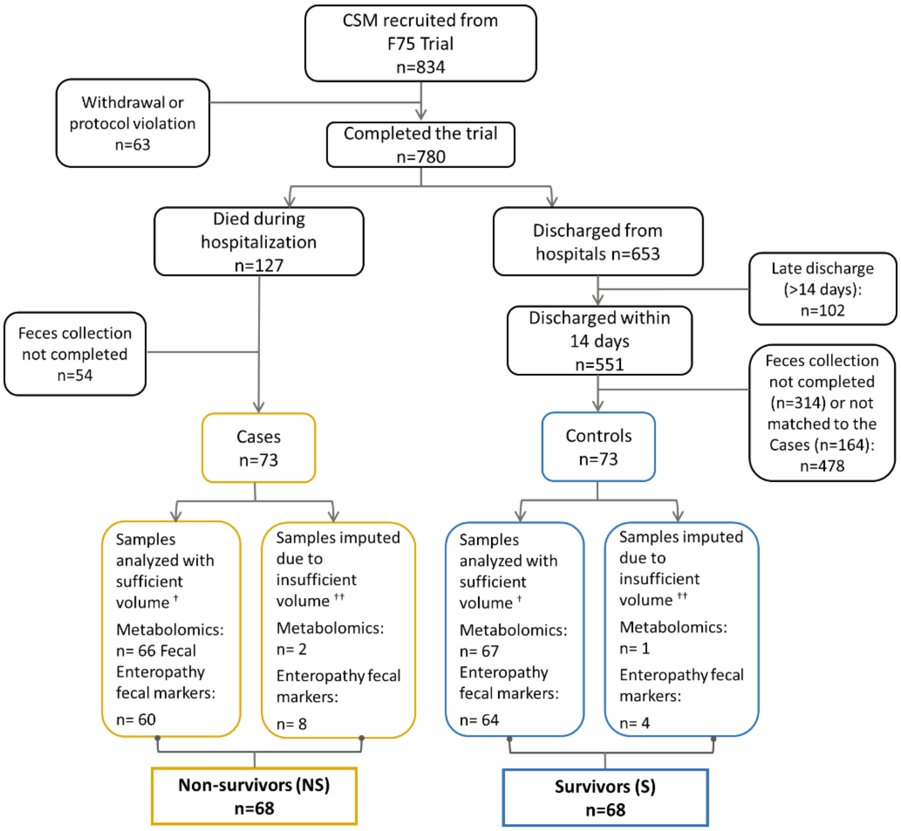

Supplementary Figure 1. Sample selection of the matched case-control study.

† 66 out of 73 cases had sufficient volume for performing metabolomics, 67 out of 73 matched controls had sufficient volume for performing metabolomics. Among the 66 cases and 67 controls, 65 were matched pairs, while 1 case and 2 controls missed corresponding pair samples.

†† To maximize use of measured metabolomics data, 2 cases and 1 control were imputed for metabolomics, and 8 cases and 4 controls were imputed for enteropathy markers.

CSM: complicated severe malnutrition.

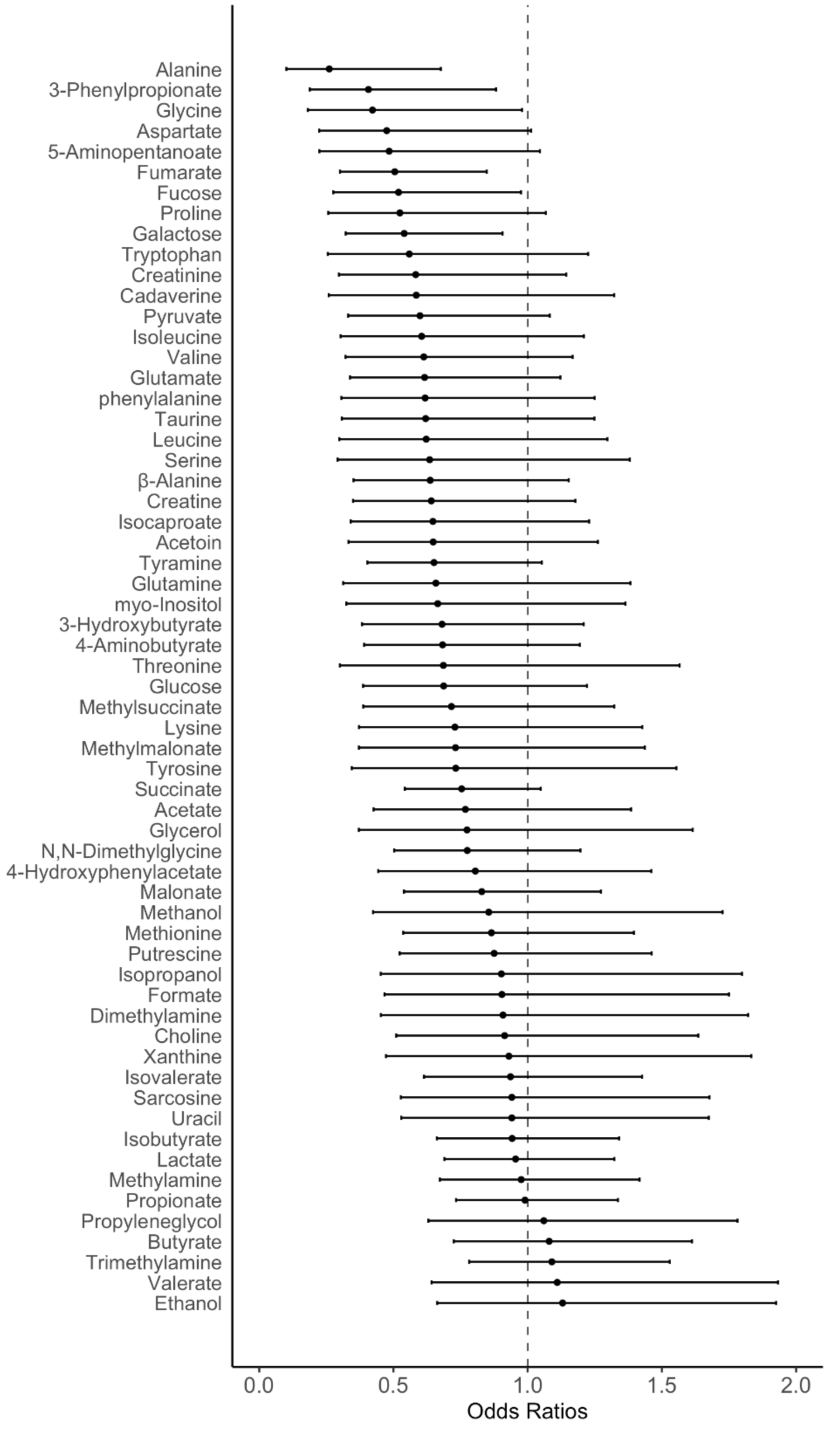

Supplementary Figure 2. Forest plot on odds ratios of mortality for a log10 unit increase in metabolite concentration. Error bars indicate 95% confidence interval.

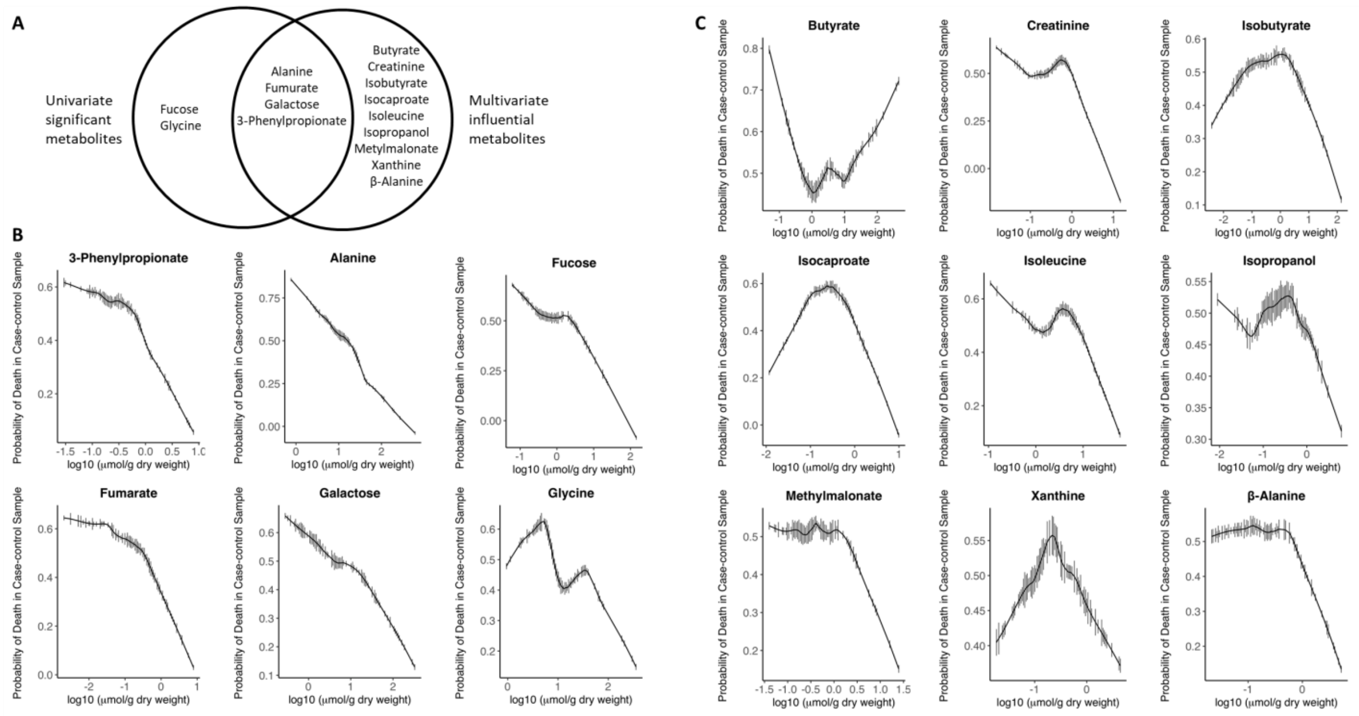

Supplementary Figure 3. (A) Venn diagram of differential metabolites identified by univariate and multivariable analyses. (B) Univariate association between differential metabolites identified by univariate analysis and the probability of death in the study sample. (C) Univariate association between differential metabolites identified by multivariable analysis and the probability of death in the study sample. In both (B) and (C), vertical bars represent the observed frequency counts of outcome for a given range of metabolite concentration, with a lowess curve fitted to depict the pattern of the association.

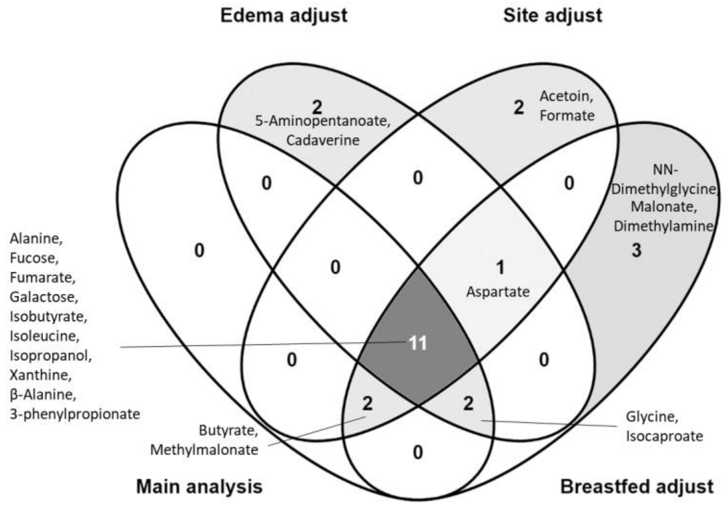

Supplementary Figure 4. Venn diagram of differential fecal metabolites identified by main analysis and sensitivity analyses.

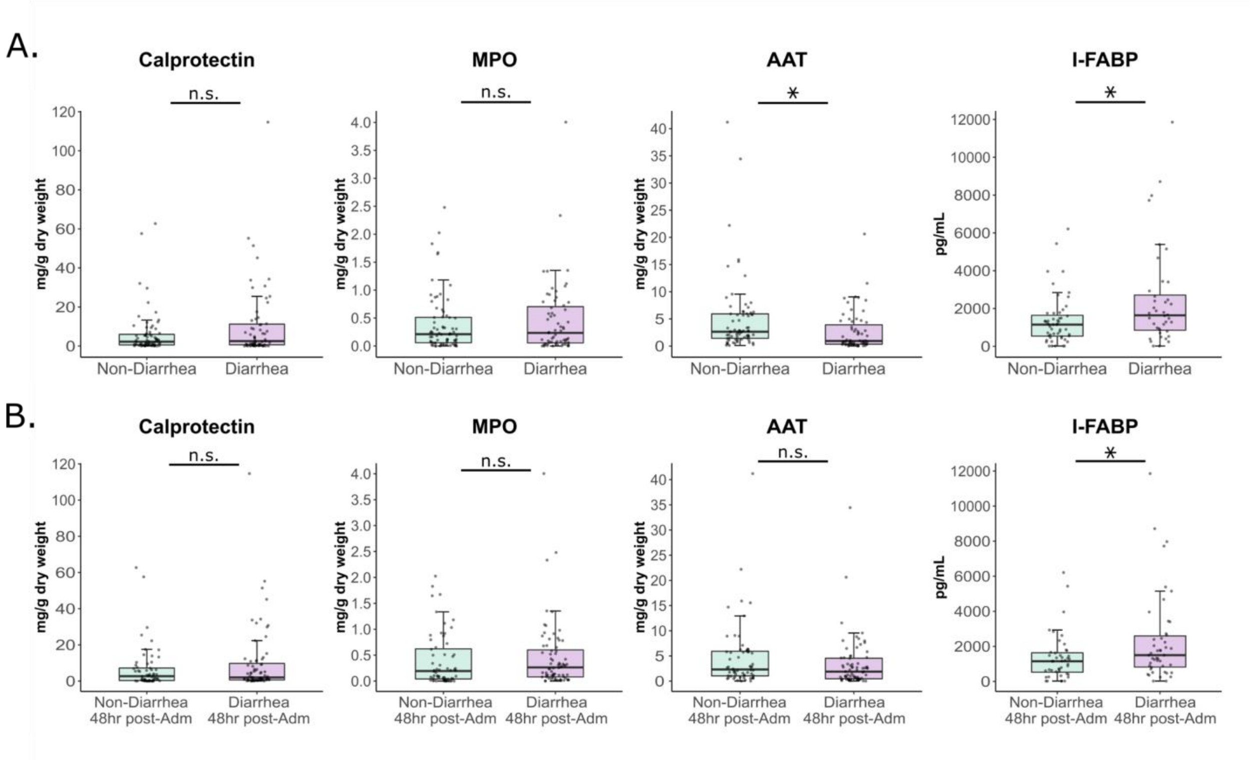

Supplementary Figure 5. Association between enteropathy marker and diarrhea at admission and 48 hours post-admission. (A) History of diarrhea reported by caregiver at admission: Calprotectin (P=0.12), Myeloperoxidase (MPO, P=0.51), Alpha-1 antitrypsin (AAT, P=0.03), Intestinal fatty acid binding protein (I-FABP) (P=0.02). (B) Presence or absence of diarrhea recorded within 48 hours post-admission (post-Adm). Calprotectin (P=0.33), MPO (P=0.57), AAT (P=0.20), I-FABP (P=0.04). Each boxplot shows the median (center line), IQR (box limits), and data points with whiskers showing 1.5 times IQR. * P<0.05, n.s. P>0.05.

Supplementary References:

1. Ritz, C., Baty, F., Streibig, J.C. & Gerhard, D. Dose-Response Analysis Using R. *Plos One*. **10,** e0146021 (2015).
2. Kuhn, M. Building Predictive Models in R Using the caret Package. *J. Stat. Sofw.* **28**, 1-26 (2008).
3. Saccenti, E., Hoefsloot, H.C., Smilde, A.K., Westerhuis, J.A. & Hendriks, M. Reflections on univariate and multivariate analysis of metabolomics data. *Metabolomics* **10**, 361-374 (2014).
4. Abram, S.V. *et al*. Bootstrap Enhanced Penalized Regression for Variable Selection with Neuroimaging Data. *Frontiers in Neuroscience.* **10**, 344 (2016).
5. Rohart, F., Gautier, B., Singh, A. & Le Cao, K-A. mixOmics: An R package for 'omics feature selection and multiple data integration. *Plos Computational Biology*. **13,** e1005752 (2017).
6. Westerhuis, J.A., van Velzen, E.J.J., Hoefsloot, H.C.J. & Smilde, A.K. Multivariate paired data analysis: multilevel PLSDA versus OPLSDA. *Metabolomics*. **6**, 119-128 (2010).
7. Szymanska, E., Saccenti, E., Smilde, A.K. & Westerhuis, J.A. Double-check: validation of diagnostic statistics for PLS-DA models in metabolomics studies. *Metabolomics*. **8**, S3-S16 (2012).
