# Supplementary material for: Intestinal disturbances associated with mortality of children with complicated severe malnutrition
Source: Commun Med (Lond). 2023 Sep 29;3:128. doi: 10.1038/s43856-023-00355-0 (PMC10541881; doi:10.1038/s43856-023-00355-0)
Supplement: Supplementary file 2 — Description of Supplementary Materials [file 43856_2023_355_MOESM2_ESM.docx]

**Description of Additional Supplementary Files**

**File name:** Supplementary Data 1

**Description:** Fecal metabolite detection.

**File name:** Supplementary Data 2

**Description:** Univariate results for fecal metabolomics.

**File name:** Supplementary Data 3

**Description:** Source data for the main figures in this manuscript.
