# Supplementary material for: Intestinal disturbances associated with mortality of children with complicated severe malnutrition
Source: Commun Med (Lond). 2023 Sep 29;3:128. doi: 10.1038/s43856-023-00355-0 (PMC10541881; doi:10.1038/s43856-023-00355-0)
Supplement: Supplementary file 6 — Reporting Summary [file 43856_2023_355_MOESM6_ESM.pdf]

nature portfolio

Corresponding author(s): Robert Bandsma

Last updated by author(s): Aug 20, 2023

Reporting Summary

Nature Portfolio wishes to improve the reproducibility of the work that we publish. This form provides structure for consistency and transparency in reporting. For further information on Nature Portfolio policies, see our Editorial Policies and the Editorial Policy Checklist.

Statistics

For all statistical analyses, confirm that the following items are present in the figure legend, table legend, main text, or Methods section.

n/a	Confirmed	
☐	☒	The exact sample size (n) for each experimental group/condition, given as a discrete number and unit of measurement
☐	☒	A statement on whether measurements were taken from distinct samples or whether the same sample was measured repeatedly
☐	☒	The statistical test(s) used AND whether they are one- or two-sided<br>*Only common tests should be described solely by name; describe more complex techniques in the Methods section.*
☐	☒	A description of all covariates tested
☐	☒	A description of any assumptions or corrections, such as tests of normality and adjustment for multiple comparisons
☐	☒	A full description of the statistical parameters including central tendency (e.g. means) or other basic estimates (e.g. regression coefficient) AND variation (e.g. standard deviation) or associated estimates of uncertainty (e.g. confidence intervals)
☐	☒	For null hypothesis testing, the test statistic (e.g. F, t, r) with confidence intervals, effect sizes, degrees of freedom and P value noted<br>*Give P values as exact values whenever suitable.*
☒	☐	For Bayesian analysis, information on the choice of priors and Markov chain Monte Carlo settings
☒	☐	For hierarchical and complex designs, identification of the appropriate level for tests and full reporting of outcomes
☐	☒	Estimates of effect sizes (e.g. Cohen's d, Pearson's r), indicating how they were calculated

Our web collection on statistics for biologists contains articles on many of the points above.

Software and code

Policy information about availability of computer code

Data collection	Chenomx NMR Suite Professional Software package v8.1
Data analysis	R 3.6.0, R packages: 'drc', 'Caret', 'mixOmics', 'glmnet', 'Hmisc', 'plspm'.

For manuscripts utilizing custom algorithms or software that are central to the research but not yet described in published literature, software must be made available to editors and reviewers. We strongly encourage code deposition in a community repository (e.g. GitHub). See the Nature Portfolio guidelines for submitting code & software for further information.

Data

Policy information about availability of data

All manuscripts must include a data availability statement. This statement should provide the following information, where applicable:

- Accession codes, unique identifiers, or web links for publicly available datasets
- A description of any restrictions on data availability
- For clinical datasets or third party data, please ensure that the statement adheres to our policy

All data supporting the findings of the study are in the main text, Supplementary Information, and public data repository. Supplementary Data 3 contains source data for the main figures in this manuscript. The fecal metabolomics and enteropathy marker data were deposited into the KEMRI-Wellcome data repository on the Harvard Dataverse under https://doi.org/10.7910/DVN/I4EYDR. The clinical data of the parent trial and the systemic data are accessible from the same repository under https://doi.org/10.7910/DVN/N4RISX and https://doi.org/10.7910/DVN/GI8YL9. All other data are available from the corresponding author on reasonable request.

Research involving human participants, their data, or biological material

Policy information about studies with human participants or human data. See also policy information about sex, gender (identity/presentation), and sexual orientation and race, ethnicity and racism.

Reporting on sex and gender	The distribution of sex (determined based on biological attributes) in our study cohort was reported within the paper. The study sample had almost equal distribution of male (52%) and female (48%). Sex-based analysis was not performed. Our study population was among young children with an average age of 2 years, where sex was not associated with mortality outcome and it is not likely to be associated with our exposures of interest.
Reporting on race, ethnicity, or other socially relevant groupings	The study did not report socially relevant groupings of study participants.
Population characteristics	This was a nested case-control study among children with complicated severe malnutrition enrolled to a multicenter randomized controlled trial (NCT02246296) in conducted Kenya and Malawi. Children with complicated severe malnutrition are defined as those with MUAC<11.5 cm, WHZ<-3 if aged less than 60 months, BMI-for-age Z-score<-3 if aged 60 months or older, or oedematous malnutrition, and had medical complications or failed an appetite test according to the WHO guidelines. This case-control study used fecal samples collected from participants on admission (before randomization and treatment initiation). All available samples collected from patients who died during hospitalization (n=68) were included, which represents 54% of all death cases in the parent trial. Each case was matched to a control sample with a similar profile of age, wasting and HIV status among patients who survived and were discharged within 14 days of admission.
Recruitment	No recruitment was performed for this study.
Ethics oversight	Ethical approval was obtained from the College of Medicine Research and Ethics Committee of the University of Malawi, the KEMRI Scientific Ethical Review Committee, Kenya, the Oxford Tropical Research Ethics Committee, and the Hospital for Sick Children, Toronto, Canada. The trial sponsor was the University of Oxford. Informed consent was obtained from parents or caregivers prior to enrollment of all study participants.

Note that full information on the approval of the study protocol must also be provided in the manuscript.

Field-specific reporting

Please select the one below that is the best fit for your research. If you are not sure, read the appropriate sections before making your selection.

[x] Life sciences [ ] Behavioural & social sciences [ ] Ecological, evolutionary & environmental sciences

For a reference copy of the document with all sections, see nature.com/documents/nr-reporting-summary-flat.pdf

Life sciences study design

All studies must disclose on these points even when the disclosure is negative.

Sample size	No sample size calculation was performed prior to the study. Sample size was limited and determined by the number of patients who died with sample available from the parent trial.
Data exclusions	Patients with late discharges (>14 days of hospital stay) were excluded from the selection of controls as they likely represented a different group of patients (e.g. chronic illnesses, sicker etc.) than survivors with a shorter length of stay, and biochemical differences at the sampling time point (admission) is not likely to have a substantial impact on their outcome 2-weeks after admission.
Replication	Due to the restriction of sample volume, technical replication of the samples in the metabolomic and ELISA assays was not performed.
Randomization	Samples were randomized for each laboratory step.
Blinding	Each sample was assigned to a coded ID such that all laboratory procedures were blinded to the identity and the grouping (i.e. case, control)

of each sample.

Reporting for specific materials, systems and methods

We require information from authors about some types of materials, experimental systems and methods used in many studies. Here, indicate whether each material, system or method listed is relevant to your study. If you are not sure if a list item applies to your research, read the appropriate section before selecting a response.

Materials & experimental systems

n/a	Involved in the study
☐	☒ Antibodies
☒	☐ Eukaryotic cell lines
☒	☐ Palaeontology and archaeology
☒	☐ Animals and other organisms
☐	☒ Clinical data
☒	☐ Dual use research of concern
☒	☐ Plants

Methods

n/a	Involved in the study
☒	☐ ChIP-seq
☒	☐ Flow cytometry
☒	☐ MRI-based neuroimaging

Antibodies

Antibodies used	Calprotectin Chemiluminescence ELISA: mouse monoclonal antibody against calprotectin, Catalog 80-CALPHU-CH01 (ALPCO, Salem, NH, USA);<br>Alpha-1 Antitrypsin ELISA: anti-human α1-antitrypsin antibody, Catalog 30-A1AHU-E01 (ALPCO, Salem, NH, USA);<br>IDK®myeloperoxidase (MPO) ELISA: mouse monoclonal anti-MPO antibody, Catalog 30-6630 (ALPCO, Salem, NH, USA);<br>I-FABP, Human, ELISA: anti human I-FABP antibody, Catalog HK406 (Hycult Biotech, Uden, Netherlands).
Validation	Antibodies and reagents used were from commercially available kits. Precision, reproducibility and validation of the reagents are provided in the respective manufacturer's instructions for use documentation.

Clinical data

Policy information about clinical studies
All manuscripts should comply with the ICMJEguidelines for publication of clinical research and a completedCONSORT checklist must be included with all submissions.

Clinical trial registration	Parent trial of this nested case-control study: NCT02246296
Study protocol	https://clinicaltrials.gov/ct2/show/NCT02246296
Data collection	The parent trial collected in-hospital clinical data from study participants recruited from 2 hospitals in Kenya and 1 hospital in Malawi between December 2014 and December 2015.
Outcomes	The outcome measure of the nested case-control study was in-hospital mortality as recorded based on clinical data collected from the parent trial.
